# Factors Associated with Deep Surgical Site Infection Following Spinal Surgery: A Pilot Study

**DOI:** 10.7759/cureus.4377

**Published:** 2019-04-03

**Authors:** Ed S Khan, Ren Yi Kow, Khairul Bariyyah Binti M Arifin, Colin Komahen, Chooi Leng Low, Bee Chiu Lim

**Affiliations:** 1 Orthopaedics, International Islamic University Malaysia, Kuantan, MYS; 2 Orthopaedics, Hospital Tengku Ampuan Afzan, Kuantan, MYS; 3 Radiology, Hospital Tengku Ampuan Afzan, Kuantan, MYS; 4 Clinical Research, Hospital Tengku Ampuan Afzan, Kuantan, MYS

**Keywords:** surgical site infections, spinal surgery, postoperative infection, thoraco-lumbar, orthopaedic surgery

## Abstract

Introduction

Surgical site infection (SSI) is the most common healthcare-related infection in surgical patients. Patients who have undergone spinal surgeries and have contracted postoperative SSI face increased morbidity and mortality, which invariably leads to additional burden on the healthcare system and higher costs. The risk factors for the increase in SSI in patients who have undergone spinal surgery have been investigated in numerous studies but no studies have been performed in Malaysia. The aim of this pilot study is to determine the incidence and factors associated with deep SSIs in patients that have undergone spinal surgeries.

Methods

This retrospective study includes all patients who underwent spinal surgeries at Tengku Ampuan Afzan Hospital, Kuantan, from 1 January 2016 to 31 December 2017. Patients with an active spinal infection, polytrauma, and open fractures were excluded from this study. Patient characteristics and laboratory investigations were extracted to determine the risk factors for deep SSI events. Associations between SSI and risk factors were analyzed with SPSS V21.0 (IBM, Armonk, NY).

Results

The univariate analysis indicated that fracture dislocation at the thoraco-lumbar junction (p=0.008) and a history of preoperative blood product transfusion (p=0.003) were associated with deep SSI. Other factors such as age (p=0.162), gender (p=0.262), body mass index (p=0.215), smoking status (0.272), number of vertebrae involved in the surgery (p=0.837), spinal cord involvement (p=0.259), postoperative hemoglobin reduction (p=0.816), and preoperative white blood cell count (p=0.278) were not associated with deep SSI.

Conclusions

This pilot study highlights the factors associated with deep SSI in spinal surgeries. A larger study is needed to further confirm these findings.

## Introduction

Surgical site infections (SSIs) are defined as superficial, deep, or organ space infections that occur within 30 days after surgery (or within one year of implant insertion) by the Centers for Disease Control and Prevention (CDC) [1]. SSI is the most common healthcare-related infection in surgical patients [2]. The rate of postoperative SSI following spinal surgery ranges from 0.7% to 12.0% and depends on the diagnosis, surgical approach, type of instrumentation, and complexity of the procedure [3-4]. Postoperative SSIs in spinal surgeries are divided into superficial and deep SSI [5]. Superficial SSIs, which involve only the skin or subcutaneous tissue, are usually treated only by antimicrobial therapy. Conversely, deep SSIs, which involve deep fascia or muscle layers, usually require a more aggressive treatment strategy such as repeated surgical debridement and prolonged administration of antibiotics [5, 6]. This leads to prolonged hospitalizations and increased patient morbidity and mortality, resulting in additional costs and burden on the healthcare system [2-3, 7-9]. 

A number of preventive measures such as strict adherence to sterile techniques, the use of prophylactic antibiotics and the application of an antiseptic solution such as povidone prior to wound closure, have been adopted to reduce postoperative SSI in spinal surgery patients [1]. In addition, numerous studies have identified several risk factors associated with an increased risk of SSI, including advanced age, obesity, diabetes, smoking, malnutrition, prolonged surgical duration, and chronic steroid use [1,4,9-19]. However, there has been no study of the factors associated with SSI in patients from Malaysia. The aim of this pilot study was to determine the incidence and factors associated with deep SSIs in patients that have undergone spinal surgeries.

## Materials and methods

This retrospective study was performed at Tengku Ampuan Afzan Hospital, Kuantan, an 851-bed spine referral center on the east coast of Peninsular Malaysia. The spinal surgeries were performed by two different spine surgeons from Tengku Ampuan Afzan Hospital and International Islamic University Malaysia. All patients that had undergone spinal surgery at our institution from 1 January 2016 to 31 December 2017 were included in this study. Patients with active spinal infection (e.g., spinal tuberculosis, pyogenic discitis, and epidural abscess) were excluded from this study. Patients with polytrauma and those with open fractures were excluded to specifically evaluate the other risk factors [20]. 

Patient characteristics such as age, gender, body mass index (BMI), smoking status, number of vertebral levels involved, spinal cord involvement, the presence of fracture dislocation at the thoracolumbar junction, and history of preoperative blood product transfusion were extracted from the patient files. Laboratory investigations such as postoperative hemoglobin reduction and preoperative white blood cell count were also included. These factors were investigated to determine the factors associated with deep SSI events. 

A deep SSI is defined as below [5,7]: - The infection occurs within 30 days after the spinal surgery. - The infection is related to the spinal surgery and involves deep soft tissue (e.g., fascial and muscle layers). - The infection requires at least one of the following:

a) Purulent drainage from the deep incision or the organ component of the surgical site. b) Surgical debridement by a spine surgeon. c) Abscess or other evidence of infection involving deep incision upon direct examination, during reoperation or by microbiological or radiological examination. d) Diagnosis of a deep SSI by the attending spine surgeon.

**Figure 1:**
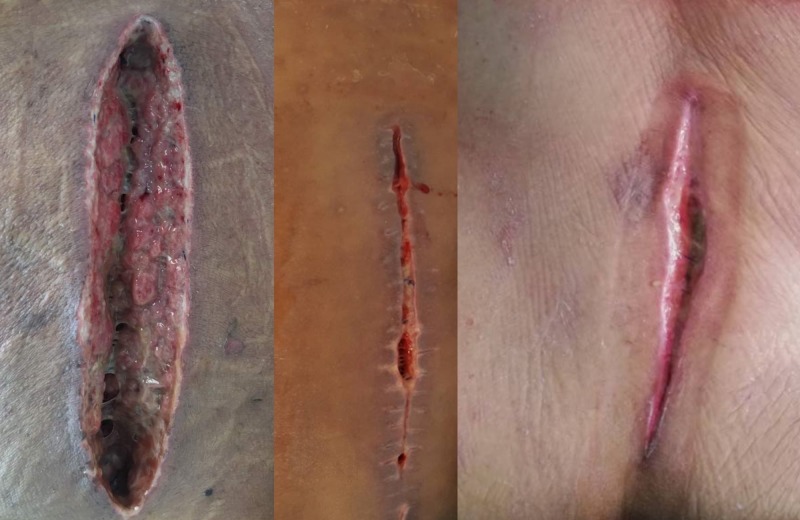
The wound condition of three different patients with an infected wound after spinal surgery (superior – cranial, inferior – caudal).

Examples of the wound conditions of different patients with an infected wound after spinal surgery are shown in Figure 1.

The associations between SSI and the risk factors were analyzed with SPSS V21.0 (IBM, Armonk, NY). Continuous variables such as age, BMI, number of vertebral levels involvement, hemoglobin reduction, and white blood cell count were compared by using a non-parametric test (Mann-Whitney) whereas categorical variables such as gender, smoking status, spinal cord involvement, fracture dislocation at the thoracolumbar junction, and history of preoperative blood product transfusion were analyzed by Fisher’s exact test. Based on the univariate analysis, any variable with a p-value of <0.05 was considered a factor associated with deep SSI events. In patients that required postoperative wound debridement, intraoperative tissue cultures were taken to determine the causative pathogens of SSI.

## Results

During the investigated period, 24 eligible patients met the inclusion criteria. Two-thirds of the patients were men (66.7%, n=16). The median age was 43 years (interquartile range 29). Four (17%) out of the 24 patients developed postoperative deep SSI that required surgical debridement. The univariate analysis found that fracture dislocation at the thoracolumbar junction (p=0.008) and a history of preoperative blood product transfusion (p=0.003) were associated with deep SSI (Table 1).

**Table 1:** Univariate analysis of patient-related and trauma-related factors associated with deep surgical site infection using Fisher’s exact test.

Variables		Non-infected n (%)	Infected n (%)	p-value
Gender	Male	12 (75%)	4 (25%)	0.262
	Female	8 (100%)	0 (0%)	
Smoking	Yes	7 (70%)	3 (30%)	0.272
	No	13 (92.9%)	1 (7.1%)	
Spinal cord involvement	Yes	11 (73.3%)	4 (26.7%)	0.259
	No	9 (100%)	0 (0%)	
Thoracolumbar junction fracture dislocation	Yes	1 (25%)	3 (75%)	0.008
	No	19 (95%)	1 (5%)	
Preoperative blood transfusion	Yes	3 (42.9%)	4 (57.1%)	0.003
	No	17 (100%)	0 (0%)	

Other factors such as age (p=0.162), gender (p=0.262), BMI (p=0.486), smoking status (0.272), number of vertebral levels involvement (p=0.298), spinal cord involvement (p=0.259), postoperative hemoglobin reduction (p=0.371), and preoperative white blood cell count (p=0.364) were not associated with deep SSI (Tables 1-2).

**Table 2:** Univariate analysis of patient-related and trauma-related factors associated with deep surgical site infection using Mann Whitney test.

Variables	Non-infected Median (Interquatile range)	Infected Median (Interquatile range)	p-value
Age (years)*	43.5(IQR 32)	41(IQR 23)	0.162
BMI (kg/m²)*	26.52(7.16)	22.27(5.80)	0.215
Number of vertebral level involved*	4.0(2.0)	4.0(1.0)	0.837
Haemoglobin reduction (g/dL)*	1.80(2.0)	1.30(3.3)	0.816
White blood cell count (x 10^9^/L)*	9.90(4.87)	8.12(5.56)	0.278

Among the patients that underwent postoperative wound debridement, all of them had deep SSI within one month postoperation. They were all treated with one or a combination of debridement and implant retention as well as antimicrobial therapy. Microbiological examination showed that there was no growth from the tissue culture in one patient (25%), a monomicrobial tissue culture in two patients (50%), and a polymicrobial tissue culture in one patient (25%). In the monomicrobial tissue culture, the causative pathogens were Staphylococcus aureus or Acinetobacter baumannii (Table 3). 

**Table 3:** The causative pathogens isolated from the intraoperative tissue culture.

Tissue cultures	Number of samples
No growth	1
Staphylococcus aureus	1
Acinetobacter baumannii	1
Polymicrobial	1

## Discussion

The overall rate of deep SSI following spinal surgeries was 17% in our setting, which was considered high compared with other studies [3]. In our setting, we strictly adhered to sterile techniques and advocated the use of prophylactic intravenous antibiotics (ceftriaxone) in all patients. However, we did not routinely use a topical antibiotic such as vancomycin at the wound prior to closure. The application of intraoperative topical vancomycin has been shown to reduce the infection rate in patients that undergo cervical and thoracolumbar fusion [21,22]. However, the routine use of intraoperative vancomycin in the wound bed could lead to an increase in gram-negative or polymicrobial spinal infections, which are often more difficult to treat. Therefore, it should be used with caution and only for high-risk patients on a case-by-case basis [23]. In addition, high-end antibiotics such as vancomycin, if not used judiciously, could lead to the emergence of vancomycin-resistant pathogens. The risk factors for SSI that we identified in our setting indicate that we should exercise extra precaution such as the use of topical vancomycin at the wound bed prior to closure to reduce the occurrence of SSI. 

Factors associated with deep surgical site infection in spinal surgery

Age and Gender

Consistent with other studies, age (p=0.162) and gender (p=0.262) were not risk factors for postoperative deep SSI following spinal surgery [13-16,18,24].

Body Mass Index

Obesity has been reported to be a risk factor for postoperative SSI after spinal surgeries. Thick layers of adipose tissue can contribute to the dead space in the surgical wound upon closure [17]. Furthermore, a larger operative field is needed for access to the spine, resulting in extensive soft tissue manipulation and retraction [17]. This could result in the formation of a large seroma and prolonged wound drainage [9]. In addition to the poor vascular supply in obese patients, the postoperative wound can be colonized by bacteria, which subsequently leads to SSI [17]. However, we found no such association in our study as there was no significant difference (p=0.215) in the BMI of patients with SSI (mean: 23.41kg/m²) and the patients with no SSI (mean: 26.12kg/m²). 

Smoking 

Previous studies have reported contradicting results with regard to smoking status as a risk factor for SSI. Veeravagu et al. found no association between smoking and the risk of postoperative spinal wound infection [18]. Conversely, Schimmel et al. reported that smoking was a risk factor for developing deep SSI after spinal fusion [7]. We found that smoking was not a risk factor for developing postoperative SSI (p=0.272).

Number of Vertebral Levels Involved 

Olsen et al. reported that surgeries involving seven or more intervertebral levels were associated with a higher risk of SSI, compared to those which involved only one intervertebral level [25]. This indicated that more extensive corrective surgery is associated with a higher risk of SSI. However, we found no association between the number of vertebral levels involved and SSI (p=0.837), as our centre did not perform any surgery involving seven or more intervertebral levels during the study period. 

Spinal Cord Involvement 

Olsen et al. found that incontinence was associated with an increased risk of SSI [19]. Similarly, Koutsoumbelis et al. found that a dural tear was associated with an increased risk of SSI [17]. A dural tear enables the cerebral spinal fluid to enter the epidural space, leading to the mixing of two separate compartments, which subsequently increases the risk of infection. Besides that, a dural tear also indirectly increases the risk of infection as the surgery tends to be prolonged and the patient is bed-bound for a longer duration [17]. However, we found no association between spinal cord involvement and increased risk of infection (p=0.259). In patients with a suspected dural tear, we tend to minimize violation to the surrounding soft tissue to create a bloodless surgical field, which might compensate for the risk of prolonged surgery contributing to SSI. 

Presence of Fracture Dislocation at the Thoracolumbar Junction 

Studies have shown that spinal surgeries performed in the thoracic and lumbar region have a significantly higher risk of SSI than spinal surgeries performed in the cervical region [25,26]. Similarly, we found that the presence of fracture dislocation at the thoracolumbar junction was associated with the development of postoperative SSI (p=0.008, OR=9.19). Patients that underwent surgical fixation of the fracture dislocation at the thoracolumbar junction were nine times more likely to contract a postoperative SSI compared to other patients. We postulate that extensive soft tissue violation during the initial trauma and subsequent corrective surgery are responsible for the increased risk of developing postoperative SSI. 

History of Preoperative Blood Product Transfusion and Postoperative Hemoglobin Reduction

Previous studies have demonstrated that postoperative anemia is associated with an increase in the SSI rate [2]. This was related to a more extensive and prolonged surgery, which carries a higher risk of infection [2]. Although we did not find an association between postoperative hemoglobin reduction (p=0.816) and deep SSI, it could be masked by the preoperative blood transfusion because a history of preoperative blood product transfusion (p=0.003, OR=12.07) was associated with a higher risk of infection. Olsen et al. also reported that the transfusion of blood products such as packed blood cells or platelets was a risk factor for developing SSI [25]. Patients that required preoperative blood product transfusion had a 12 times higher risk of contracting postoperative deep SSI. This could be owing to a significant loss of blood during the initial trauma to the soft tissue and vertebrae, which leads to a higher risk of contracting postoperative deep SSI. 

Preoperative White Blood Bell Count

Preoperative white blood cell count (p=0.278) was not associated with deep SSI in our study because we excluded patients with active spinal infection. This result suggested that SSI is an ongoing process that happens during and after the spinal surgery. 

Microbiology Profile

A previous study by Ghobrial et al. showed a higher percentage of gram-positive causative pathogens (86.3%) in spine surgery patients with SSI [23]. In our case, half of the causative pathogens were gram-negative or polymicrobial pathogens, which suggested that it may be a hospital-acquired infection. 

Study limitations

Our study has several limitations. A limited number of patients were recruited in a retrospective manner, which may not be representative of the entire Malaysian population. We were unable to recruit more patients owing to missing files for patients that had undergone surgeries prior to 2016. Furthermore, we were only able to retrieve limited data retrospectively. Therefore, some of the variables such as nutrition status, surgical duration, and immuno-compromise status, which have the potential to influence the development of SSI, were not included. Because there was no such data available for patients from the east coast of Peninsular Malaysia, this pilot study serves as a guide for a larger prospective study in the future.

## Conclusions

In this pilot study, we identified a high rate of deep SSI among patients that underwent spinal surgery at Tengku Ampuan Afzan Hospital, Kuantan, Malaysia, between 1 January 2016 and 31 December 2017. Fracture dislocation at the thoracolumbar junction and preoperative blood product transfusion were identified as factors associated with deep SSI. A larger prospective study is needed to confirm the findings.
